# Vaccination Coverage of the Elderly in Greece: A Cross-Sectional Nationwide Study

**DOI:** 10.1155/2020/5459793

**Published:** 2020-06-27

**Authors:** Dimitrios Papagiannis, Georgios Rachiotis, Anargiros Mariolis, Efterpi Zafiriou, Konstantinos I. Gourgoulianis

**Affiliations:** ^1^Department of Nursing, School of Health Sciences, University of Thessaly, Larissa, Greece; ^2^Department of Hygiene and Epidemiology, School of Health Science, Medical Faculty, University of Thessaly, Larissa, Greece; ^3^National Health System, Areopolis Health Center, Areopoli, Greece; ^4^Department of Dermatology, School of Health Sciences, Medical Faculty, University of Thessaly, Larissa, Greece; ^5^Department of Respiratory Medicine, School of Health Sciences, Medical Faculty, University of Thessaly, Larissa, Greece

## Abstract

Vaccines are important for older adults, and the morbidity and mortality of vaccine-preventable diseases among older adults are high. There are limited data on vaccination coverage among elderly people in Greece. The aim of this observational study was to record the vaccination coverage for vaccines recommended by the National Vaccination Program in Greece for the elderly people ≥60 years old. Two hundred general practitioners (GPs) around the country from the primary healthcare system were invited to “participate,” and one hundred fifty from them participated in the present study. The GPs were selected using geographically stratified random sampling methodology. Two thousand and seventy-two participants participated in the present study: of which, 1043 were males and 1029 were females. The mean age of the participants was 73.3 years, and 83% vaccination coverage for flu vaccine, 49.5% for conjugate pneumococcal vaccine, and 23.5% for polysaccharide pneumococcal vaccine were recorded. In addition, the vaccination coverage for herpes zoster vaccine was 20%, while very low percentages were recorded for diphtheria, tetanus, pertussis, and polio vaccine for adults. We found significant gaps in vaccination coverage, especially with regard to pneumococcal, herpes zoster, and tetanus. On the contrary, influenza vaccination coverage was satisfactory.

## 1. Introduction

Vaccines are crucially important for older adults (elderly people). The immune system weakens with ageing exhibiting a progressive functional decline referred to as immunosenescence that collectively results in diminished humoral and cellular immune responses, and it can be more difficult to fight off infections [1–3]. Older adults are at high risk of diseases such as the flu, pneumonia, and shingles and of complications that can lead to hospitalization, long-term illness, and even death [4]. Vaccines against influenza, *S. pneumoniae*, and herpes zoster are available for the elderly and vaccines that are used throughout adulthood, such as tetanus, diphtheria, and pertussis, may be also relevant for the elderly people [5].

The vaccination recommendations for adults and the elderly differ from country to country. Vaccination coverage also differs between countries, and data are very difficult to obtain, as many countries do not have centralized databases collecting this information. The WHO goal of 75% influenza vaccination coverage for older adults (>65 years) until 2014/2015 was not reached by most countries [6]. Multimorbidity including chronic obstructive pulmonary disease (COPD), cancer, cardiovascular diseases, diabetes, and obesity increases with age. Furthermore, the raise in the prevalence of multimorbidity could be a risk factor for the occurrence of vaccine-preventable diseases [7, 8]. An improvement in vaccination coverage for the elderly can decrease the burden of these chronic conditions by reducing morbidity, hospital admissions, health costs, and mortality rates. On the other hand, high vaccination coverage in the population may further reduce morbidity and mortality of vaccine-preventable diseases in adults through the mechanism of herd immunity [9]. Furthermore, there is some epidemiological evidence which indicates the cost-effectiveness of influenza and pneumococcal vaccination [10].

The current health policies on immunizations rely on the transition from infants and young adults' vaccination programs to vaccination programs for all ages and all groups and specially to the elderly. Wu et al., in a review of vaccination programs for adults, showed that only 38.7% of 31 developed countries had comprehensive adult vaccination schedules, with significant variations in terms of the number of vaccines or vaccine components. The most frequently recommended vaccines for adults are seasonal influenza, hepatitis B, pneumococcal, tetanus, and diphtheria [11]. Cassimos et al. in another study for vaccination programs in Europe report considerable differences between countries in terms of the number of vaccinations, target populations, and frame of implementation recommended or mandatory vaccinations [12]. In Greece, vaccinations are financially supported by the National Insurance system and the Greek National Immunization Program offers free of charge the vaccines in infants, some special groups, e.g., health professionals, students, and immunocompromised patients, and to the healthy adults. From 2011, influenza and herpes zoster vaccines have been recommended in Greece for everyone over 65 and 60 years of age accordingly, and the pneumococcal disease vaccine has been recommended for those aged 65 and older. An adult catch-up program was implemented for Tdap (tetanus, diphtheria and acellular pertussis or diphtheria tetanus or tetanus monovalent vaccine for adults as a booster dose every 10 years of life. Vaccination against diphtheria, tetanus, and pertussis is recommended for all adults. This is also the case for tetanus monovalent vaccine [13].

To the best of our knowledge, there are no published data on the vaccination coverage among the elderly in Greece. Consequently, the aim of our study was to investigate the vaccination coverage among elderly people in Greece. Special emphasis was given to flu, pneumococcal, and herpes zoster vaccines.

## 2. Sampling Methods

In this study, we collected data from a nationwide sample of general practitioners (GPs) in Greece. Greece consists of 13 administrative regions, nine of which belong to mainland Greece (Anatoliki Makedonia/Thraki, Kentriki Makedonia, Dytiki Makedonia, Thessalia, Ipeiros, Dytiki Ellada, Sterea Ellada, Peloponnisos, and Attiki) and four insular regions (Ionia Nisia, Voreio Aigaio, Notio Aigaio, and Kriti). These regions correspond to the NUTS 2 level. The GPs were selected using a nonprobability sampling method (quota sampling). In Quota Sampling, the groups in the sample are proportional to the groups in the population. Consequently, the sample of GPs was proportional to the population size of each region. We invite 200 general practitioners from 12 out of the 13 regions of Greece (the region of Voreio Aigaio was not included) to participate in this study, and 150 GPs participated (response rate: 75%). The GPs administered, through a face-to-face interview, a questionnaire to the patients (adults >60 years old). The questionnaire included questions on vaccination history for five vaccines recommended by the Greek National Immunization Program. The sample of patients was convenient. The study was conducted from March to September 2019.

### 2.1. Questionnaire

A questionnaire was completed by the GPs following the informed consent of the patient. The questionnaire included sociodemographic information (sex, age, ethnicity, and vaccination status for the vaccines recommended by the National Vaccination Program for the Elderly). In particular, the questionnaire included questions on the number of doses of vaccinations for the following vaccines (influenza, pneumococcal, herpes zoster, tetanus, diphtheria, pertussis, and polio). In addition, the general practitioners recorded the comorbidities of the participants. The questionnaire was pretested among 8 GPs through telephone contacts in order to assimilate survey conditions and to ensure accuracy and consistency of the questions. The protocol of the study was approved by the Ethical Committee, number 545/25-1-2019, of the Technological Educational Institute of Thessaly.

### 2.2. Statistical Analysis

Data were entered into an Excel database and were tabulated. Absolute (*n*) and relative frequencies (%) were presented for qualitative variables, and means (with standard deviations) were used for continuous variables. In addition, 95% confidence intervals (95% CIs) were calculated for proportions by the use of OpenEpi.

## 3. Results

In total, 2072 subjects participated in the study. The demographic characteristics of the 2072 participants who participated in this study were as follows: 1043 (50.3%) males 1029 (49.7%) females; the mean age of the participants was 73.3 years, and the age range was from sixty to ninety-five years. The vast majority of the participants were of Greece ethnicity followed by the Cypriot, Albanian, Bulgarian, English, German, and finally 6.3% was of unknown ethnicity (Table 1). One thousand seven hundred and eighty (83%) of the patients had been vaccinated with the flu vaccine at the previous period. The vaccination coverage for conjugate pneumococcal vaccine (PCV-10, 13) was 49.5% and 23.5% for pneumococcal polysaccharide vaccine (PPSV-23). Four hundred and twenty-nine persons, 20%, of the total sample had received the herpes zoster vaccine. The presentence of vaccination coverage for the diphtheria, tetanus, pertussis, and polio was very low. The vaccination coverage for Td vax adult vaccine was 0.30%, for the Tdap-IPV was 0.01%, and for the monovalent vaccine of tetanus was only 0.30%. Regarding HBV vaccination, only sixty-six of the participants (0.3%) have reported vaccination with three doses for hepatitis B vaccine (Table 2). The distribution of patients according to risk medical conditions was as follows: one hundred and nine had asthma (5.5%), six hundred and eighty-four of the participants had diabetes mellitus (34%), two hundred and eighty-seven had (14%) chronic obstructive pulmonary disease (COPD), and six hundred and eighty-eight of patients (34%) had heart diseases, followed by 6.5% of patients with cancer, 3% with immunodeficiency conditions, and finally 3% with kidney diseases (Table 3).

## 4. Discussion

In this study, which is the first in Greece investigating the vaccination coverage for elderly people >60, we found significant gaps in vaccination coverage. In particular, the vaccination coverage varied greatly from 83% for flu vaccine to 0.1% for tetanus vaccine.

### 4.1. Influenza Vaccine

There is epidemiological evidence that influenza was associated with an excess of all cause's mortality among individuals ≥65 years of age in 14 European countries [14]. Furthermore, in the WHO European Region, the vaccination coverage against influenza remains low among people aged 65 years or above [15, 16]. The National Vaccination Program in Greece also recommend the annual vaccination with flu vaccine for people up to 60 years [13]. In the present study, the vaccination coverage for influenza was 83%. This rate is well above the European median vaccination coverage of 34.4% in 2014-2015.

### 4.2. Pneumococcal Vaccine

The prevalence of antibiotic-resistant *S. pneumoniae* has increased over the last decade. Older age is a strong determinant of pneumococcal mortality. Adults aged ≥60 years comprise a high case-fatality rate group (≥15%). Mortality increases substantially with age and is 2- to 5-fold higher in adults with underlying diseases than in healthier older adults. It has been reported that, in elderly people with chronic illness, the pneumococcal polysaccharide vaccine (PPSV) may reduce hospitalization during the influenza season [17]. The National Vaccination Program in Greece recommend the vaccination with pneumococcal 23-valent polysaccharide, 10-valent conjugate, and 13-valent conjugate vaccine for the people up to 65 years and the special groups, e.g., patients with heart failure or diabetes. We reported a vaccination coverage of 49.5% and 23.5% for conjugate and polysaccharide pneumococcal vaccine, respectively. In a large cohort of 58,589 individuals in Australia, the vaccination coverage for the elderly people was 69% for the age group of 60–65 years for conjugate pneumococcal vaccine and the vaccination was more likely among those with comorbidities [18]. The results from another local study in Catalonia, Spain, showed that 38.7% had received PPSV-23 at any time during the last 5 years and only 0.7% at all age groups from the population study had received conjugate pneumococcal vaccine (PCV-13) in the previous 5 years [19]. Another study from one region of Greece with 318 participants (56.6%) had received a flu vaccine in 2018, while 50.8% received it annually in previous years' significant difference with the present study [20].

### 4.3. Herpes Zoster Vaccine

The overall incidence of herpes zoster (HZ) increases with age among persons 80 years of age or older [21]. An increased incidence of HZ has also been associated with immunosuppression related to certain comorbidities, such as solid tumors, hematological malignancies, HIV infection, and autoimmune diseases, and to immunosuppressive treatment. Vaccination is, therefore, an attractive option to reduce the disease burden due to herpes zoster and its complications in older adults. Currently, a live attenuated herpes zoster vaccine is approved for use in adults 50 years of age or older and is recommended for immunocompetent adults ≥60 years by the National Greek Immunization Program. We found a vaccination coverage for herpes zoster of 20% for the elderly participants. Zhang et al. from the USA investigated a large cohort of insured individuals aged ≥50 years and reported that 14.5% of adults aged ≥65 years received HZ vaccine [22]. Another study from the USA showed significant state-specific variation for the herpes zoster vaccination coverage among adults aged ≥60 years with a median of 33.3% (range: 17.8%–48.8%) [23]. Vaccination against herpes zoster is recommended by the National Immunization Program for adults.

### 4.4. Diphtheria, Tetanus, Pertussis, and Polio Vaccine

There are several lines of epidemiological evidence indicating that tetanus- and diphtheria-specific antibody titles are below the cutoff levels considered to be protective for the elderly [24–26]. With respect to pertussis, there is evidence that this pathogen is associated with increased mortality among the elderly [27, 28]. A study from Italy reported that 82% of the tetanus cases between 2001 and 2010 occurred in people aged more than 65 years [29]. Regular booster shots against tetanus and diphtheria in some cases in combination with pertussis are recommended throughout adulthood in many countries, and the majority of the European countries recommend regular booster vaccinations throughout adulthood every 10 years [30]. Notably, in the present study,we report very low vaccination coverage against tetanus, and perhaps, this finding may reflect low physicians' compliance with tetanus vaccination.

### 4.5. Hepatitis B Vaccine

Hepatitis B virus infection could be a potential hazard, especially for the older long-distance travelers, and thus, HBV vaccination could be considered for older travelers [29]. The results of the present study indicate extremely low vaccination coverage for hepatitis B vaccine, and specifically, only sixty-six of the total samples of 2072 participants have to receive three doses for hepatitis B vaccine. According to the Greek National Immunization Program, vaccination against hepatitis B is a catch-up dose or doses of vaccine depending on history of vaccination and available to those over the age of 18 years.

Our results are subject to several limitations. First, the present study was questionnaire based and some information bias may have occurred. Second, we employed a nonprobability sampling method and there is a potential for selection bias to occur. Third, the convenient sampling of the patients by the GPs may have limited the external generalizability of our results.

## 5. Conclusions

In this study, which is the first in Greece investigating the vaccination coverage for elderly people >60 years, we found significant gaps in vaccination coverage, especially with regard to pneumococcal, herpes zoster, and tetanus. On the contrary, influenza vaccination coverage was satisfactory. Our results provide preliminary evidence on the status of vaccination coverage among the elderly in Greece and underline the need for initiatives for the improvement of vaccination coverage among the elderly people in Greece.

## Figures and Tables

**Table 1:** Demographical characteristics of participants.

Characteristics	Total, *N*	%
Participants	2072	100
Male	1043	50.3
Female	1029	49.7

Mean age (SD)	73.3	(27.3)

Age group (yrs)		
60–70	817/2072	39.5
70–80	834/2072	40.5
80+	421/2072	20

Ethnicity		
Greece	1822/2072	87.9
Cypriot	56/2072	2.7
Albanian	43/2072	2.1
Bulgarian	6/2072	0.3
English	2/2072	0.1
German	12/2072	0.6
Unknown	131/2072	6.3

**Table 2:** Vaccination coverage.

Vaccines	Vaccination coverage total sample			Male			Female		
	*N*	%	95% CI	*N*	%	95% CI	*N*	%	95% CI
Flu	1718/2072	83.0	(81.2–84.5)	878/1043	84.2	(82–86.3)	842/1029	81.9	(79.4–84.1)
Pneumococcus polysaccharide	480/2072	23.2	(21.4–25)	249/1043	23.9	(21.4–26.6)	232/1029	22.5	(20.1–25.2)
Pneumococcus conjugate	1029/2072	49.7	(47.5–51.8)	551/1043	52.8	(49.8–55.8)	480/1029	46.6	(43.6–49.7)
Herpes zoster	429/2072	20.7	(19–22.5)	231/1043	22.1	(19.7–24.8)	198/1029	19.2	(17–21.8)
Hepatitis B	66/2072	3.18	(2.5–4.03)						
Td vax adult	64/2072	3.1	(2.4–3.9)						
Tdap-IPV	25/2072	1.2	(0.81–1.78)						
Tetanus monovalent	62/2072	3.0	(2.3–3.8)						

**Table 3:** Distribution of patients according to risk medical conditions and vaccination status.

Medical conditions	Total patients	Vaccination status and health conditions		
		Flu	PPSV-23	PCV-10, 13
	*N* (%)	*N* (%)	*N* (%)	*N* (%)
Asthma	109/2072 (5.26)	76/109 (69.7)	29/109 (26.6)	46/109 (42.2)
Diabetes	684/2072 (33.01)	572/684 (83.6)	210/684 (30.7)	342/684 (50)
COPD	287/2072 (13.9)	265/287 (92.3)	94/287 (32.8)	191/287 (66.5)
Heart disease	688/2072 (33.2)	597/688 (86.7)	150/688 (21.8)	442/688 (64.2)
Cancer	123/2072 (5.93)	112/123 (91.1)	40/112 (35.7)	88/123 (71.5)
Immunodeficiency	62/2072 (3)	49/62 (79)	15/62 (24.2)	43/62 (69.4)
Kidney disease	61/2072 (2.94)	60/61 (98.3)	13/61 (21.3)	50/61 (82)

## Data Availability

The data used to support the findings of this study are restricted by the ethics commitee in order to protect patient privacy. Data are available from the corresponding author upon reasonable request for researchers who meet the criteria for access to confidential data.
